# Combining accelerometers and direct visual observations to detect sickness and pain in cows of different ages submitted to systemic inflammation

**DOI:** 10.1038/s41598-023-27884-x

**Published:** 2023-02-03

**Authors:** Dorothée Ledoux, Isabelle Veissier, Bruno Meunier, Valérie Gelin, Christophe Richard, Hélène Kiefer, Hélène Jammes, Gilles Foucras, Alice de Boyer des Roches

**Affiliations:** 1 Université Clermont Auvergne, INRAE, VetAgro Sup, UMR Herbivores, 63122 Saint-Genès-Champanelle, France; 2 Université Paris-Saclay, UVSQ, INRAE, BREED, 78350 Jouy-en-Josas, France; 3 UCEA, INRAE, Université Paris Saclay, 91630 Leudeville, France; 4 Ecole Nationale Vétérinaire d’Alfort, BREED, 94700 Maisons-Alfort, France; 5 IHAP, Université de Toulouse, ENVT, INRAE, 31076 Toulouse, France

**Keywords:** Animal behaviour, Animal physiology, Diseases, Infectious diseases, Signs and symptoms, Fever, Pain, Diagnostic markers, Biomedical engineering, Cytokines, Inflammation

## Abstract

Cattle suffering from inflammatory infection display sickness and pain-related behaviours. As these behaviours may be transient and last only a few hours, one may miss them. The aim of this study was to assess the benefit of combining continuous monitoring of cow behaviour via collar-attached accelerometers with direct visual observations to detect sickness and pain-related behavioural responses after a systemic inflammatory challenge (intravenous lipopolysaccharide injection) in cows of two different ages, proven by clinical, physiological and blood parameters. Twelve cloned Holstein cows (six ‘old’ cows aged 10–15 years old and six ‘young’ cows aged 6 years old) were challenged and either directly observed at five time-points from just before the lipopolysaccharide injection up to 24 h post-injection (hpi) or continuously monitored using collar-attached accelerometers in either control or challenge situations. Direct observations identified specific sickness and pain behaviours (apathy, changes in facial expression and body posture, reduced motivation to feed) expressed partially at 3 hpi and fully at 6 hpi. These signs of sickness and pain behaviours then faded, and quicker for the young cows. Accelerometers detected changes in basic activities (low ingesting, low ruminating, high inactivity) and position (high time standing up) earlier and over a longer period of time than direct observations. The combination of sensors and direct observations improved the detection of behavioural signs of sickness and pain earlier on and over the whole study period, even when direct signs were weak especially in young cows. This system could provide great benefit for better earlier animal care.

## Introduction

Cattle suffering from bacterial infection (like bronchopneumonia^1^, mastitis or metritis^2^, are the most frequent), frequently associated with sepsis, display sickness and pain-related behaviours^3–5^. Sick animals separate from conspecifics^4^ and change their basic activities: they are reluctant to move, lie down more frequently and have a lower feed intake, and their circadian rhythm of activity is altered^3,6^. Cattle experiencing pain display specific behaviours in response to the noxious stimuli (trauma or inflammation), such as antalgic postures and specific facial expressions^7,8^. These sickness and pain-related behaviours reflect the animal’s experience of discomfort—in other words its experience of poor welfare. Detecting such behavioural signs is vital in order to implement appropriate corrective measures and treatments as early as possible to restore homeostasis and improve animal welfare. In adult cows, behavioural response to sickness has been already studied after local inflammatory challenge such as experimental mastitis^9–12^ but not yet in systemic inflammation.

The changes in cow behaviours are driven by the inflammatory response to infection. Inflammation is triggered by the release of pro-inflammatory mediators such as eicosanoids and cytokines, including interleukins 1α and β (IL-1α and IL-1β), interleukin-6 (IL-6) and tumour necrosis factor α (TNF-α) that are produced at the site of infection by activated accessory immune cells (e.g. macrophages and monocytes) and operate as the most potent triggers of the inflammatory response. Cytokines induce the acute-phase response, which lasts only a few hours^13,14^. They act on the brain, causing behavioural symptoms of sickness^3,4^, endocrine responses (e.g. cortisol release^13^), and systemic pathophysiological signs (hyperthermia, decreased rumination rate, increased heart and respiratory rates, apathy, anorexia, and other specific clinical signs)^13^. Cytokines act on the liver to induce the synthesis of acute-phase proteins like haptoglobin and serum amyloid A (SAA)^13,14^. Proper characterization of the organism’s response to infection-related sickness therefore requires behavioural observations as well as an assessment of clinical signs and biological parameters like blood inflammatory biomarkers, at least during the acute phase of the response.

Current strategies in cattle breeding tend to extend the duration of the productive life of dairy cows^15^. However, genetic^16^ or others factors, such environment or age, should be taken into account. The older the cows, the more susceptible they are to disease. The less pronounced inflammatory responses in pluriparous cows compared to those of primiparous cows may explain this higher susceptibility to disease^17^. We therefore question whether sickness and pain-related behaviour, which are linked to inflammatory responses, vary with age, and whether this can affect their detection in old cows.

Because the signs of sickness and pain caused by inflammation may be transient and last for only few hours^11,18,19^, there is a high chance that they will be missed by farmers and caretakers during brief routine observations performed once or twice a day. Reliably detecting such changes would require observations of the animals throughout the day or over numerous repeated sufficiently long periods, which would pose a technical challenge. Sensor-based systems such as accelerometers^20^, which are already in widespread use on dairy farms^21^, make it possible to monitor animals continuously and in real time. Activity changes can be detected via mathematical models^20^. Changes in activity can be due to environmental factors (atmospheric conditions, human intervention) or reproductive status (oestrus, calving). For instance, exposure to heat is associated with a sharp decrease in feed intake^22^, and increased activity is typically a sign of oestrus in cattle^23^. Such activity changes should therefore be associated with more specific behavioural indicators (e.g. apathy, antalgic posture) that at present are not detected by accelerometers. There is therefore a need to combine observations from sensors with direct visual observations to refine the detection of a sick animal.

Gram-negative bacteria are prevalent cattle pathogens characterized by cell walls that feature lipopolysaccharide (LPS), the most potent pro-inflammatory molecule. When injected intravenously to animals, LPS induces similar responses to those observed in gram-negative bacterial infection, i.e. release of pro-inflammatory cytokines including TNF- α, IL-1α, IL-1β, IL-6^19,24–27^ and inflammatory proteins (haptoglobin, SAA)^19,25–28^, changes in physiological parameters (rectal temperature, heart and respiratory rate)^18,19,24,26,27,29^, and clinical signs (ocular discharge, sweat and dehydration)^18,19^ and changes in endocrine parameters including increased cortisol level^19,24,26,27,29^ for less than 24 h. After systemic inflammation induced by an intravenous bolus of LPS, calves aged 3 weeks or 5 months decreased their time spent ruminating, grooming and eating hay and increased their time spent lying and standing inactive^18^. LPS challenge is therefore an appropriate model for studying the physiological and behavioural responses of animals to systemic short-term inflammation.

The aim of this study was to assess the potential benefit of combining continuous monitoring of cow behaviour via a collar-attached accelerometers with visual direct observations to detect sickness and pain-related behavioural responses in young vs. old cows. We used the model of LPS injected intravenously to generate a systemic inflammation. We characterized the phases of the inflammation by monitoring cytokine, cortisol, and acute-phase protein concentrations and physiological parameters during a 24-h time-window corresponding to the early stress response. To reduce the variability due to individual factors, we had the opportunity to examine the sickness and pain-related behavioural responses on an outstanding group of cows with the same genetic background, born over a 15-years period. The cows were issued by somatic nuclear transfer from a single cell and that had been reared in same conditions. They were aged 6–15 years, which allowed us to identify the precise effect of age.

## Methods

### Ethics statement

In compliance with European Directive 2010/63/EU, the protocol and procedures were approved by the Ile-de-France institutional animal care and use committee (France, DGRI agreement APAFIS #11503-2017091411167913). All procedures were applied by trained staff members who performed the trial in accordance with all relevant named guidelines and regulations. The study was carried out with the ARRIVE guidelines. Endpoints were defined before the start of the experiment: in the event of rectal temperature above 41.5 °C or abnormal results of a veterinarian-led clinical examination over 24 h, a non-steroidal anti-inflammatory drug (meloxicam 0.5 mg/kg BW IV) was provided. If the endpoint was reached during CONTROL or CHALLENGE situations (see below), the data from the cow was excluded.

### Animals, housing and feeding

This study is part of a wide project deciphering the role of ageing on the response to inflammation in cattle, and more especially the methylation of the genome according to age and inflammation status (not reported here). The present study was grafted onto the same experimental design. All cows were generated by nuclear transfer using cultured ear explant-derived fibroblasts as previously described^30,31^. As somatic cloning can be performed using nuclei from frozen fibroblasts, it makes possible to generate individuals for more than 15 years using the original cell culture. Thus, the initial sample comprised 14 Holstein cows with the same genetic background: 6 cows aged 10–15 years (body weight: 693.5 ± 87.3 kg, referred to as ‘old cows’) and 8 cows aged 6 years (678.2 ± 37.9 kg, referred to as ‘young cows’).

The cows were housed in a loose-stall deep-bedded barn at the INRAE Experimental Facility at Bressonvilliers, France. The cows had been raised together since birth. The two groups, i.e. old and young cows, were housed in two face-to-face pens. The 8 ‘young’ cows were housed together with 9 contemporary (i.e. same-age, same-breed) non-cloned cows in a 18 × 28 m pen. No measurements were performed on contemporary cows. The 6 ‘old’ cows were housed in a 15.4 × 32 m pen. All cows in each pen had been together for more than 10 months before the start of the study. They were fed a corn silage, soybean meal and concentrate ration designed to meet the dietary requirements for dry cows. Feed was given once a day at 10:00 a.m. The mixed ration was pushed back toward the cows three times a day (at 2:30 p.m., and 8:30 p.m. then at around 7:30 a.m). Water was provided ad libitum.

To avoid oestrus behaviour, the study was carried out during the cows’ luteal phase. At 27 days before challenge, the cows were submitted to an ovulation synchronization protocol using a Norgestomet ear implant for 10 days simultaneously with intramuscular injections of 10 µg buserelin at the start (CRESTAR PACK, Intervet, Beaucouzé, France), then 0.5 mg prostaglandin (ESTRUMATE, Intervet, Beaucouzé, France) 8 days later, and 400 UI of eCG (SYNCRO-PART PMSG 400 UI BOVINS-OVINS-CAPRINS, Ceva Santé Animale, Libourne, France) on the day of ear-implant removal. Effective ovulation was checked at 7 days after the end of the synchronization protocol by palpation of *corpus luteum*. All cows had a visible *corpus luteum*, showing similar ovarian activity between the young and old groups.

### Experimental design and challenge

This experimental study used a longitudinal cross-over design, with individual cows serving as their own controls. Two situations were applied: CHALLENGE (after being headlocked and receiving LPS injection) vs. CONTROL (after being headlocked but not injected with LPS). There were four batches of cows, with two young cows and one old cow in Batches 1 and 2 and two young cows and two old cows in Batches 3 and 4. Cows from Batch 1 were LPS-challenged first, then cows from Batch 2 were LPS-challenged on the following day. Cows from Batch 3 and 4 were LPS-challenged three weeks later, again with one day between the two batches. Cows from Batches 1 and 2 were CHALLENGED when the cows from Batches 3 and 4 were in their CONTROL situation, and vice versa. The LPS challenge consisted in a bolus injection of LPS (0.5 μg/kg Ultrapure LPS, *E. coli* 0111:B4, InVivogen, Toulouse, France) into the jugular vein at 09:30 a.m. (± 16 min) (T0). The dose was chosen to mimic an acute inflammation with no lasting and serious impact on the animal^24,26,27^.

### Data collection

One month before the beginning of the study, the cows were fitted with collar-attached tri-axial accelerometers (AXEL sensor) commercialized by ITK/NewMedria (Chateaubourg, France). The collar was tightly attached with a buckle. During three consecutive days before any observation, the cows were handled (headlocked and clinically examined) by researchers to habituate them to the experimental procedures.

**Figure 1:**
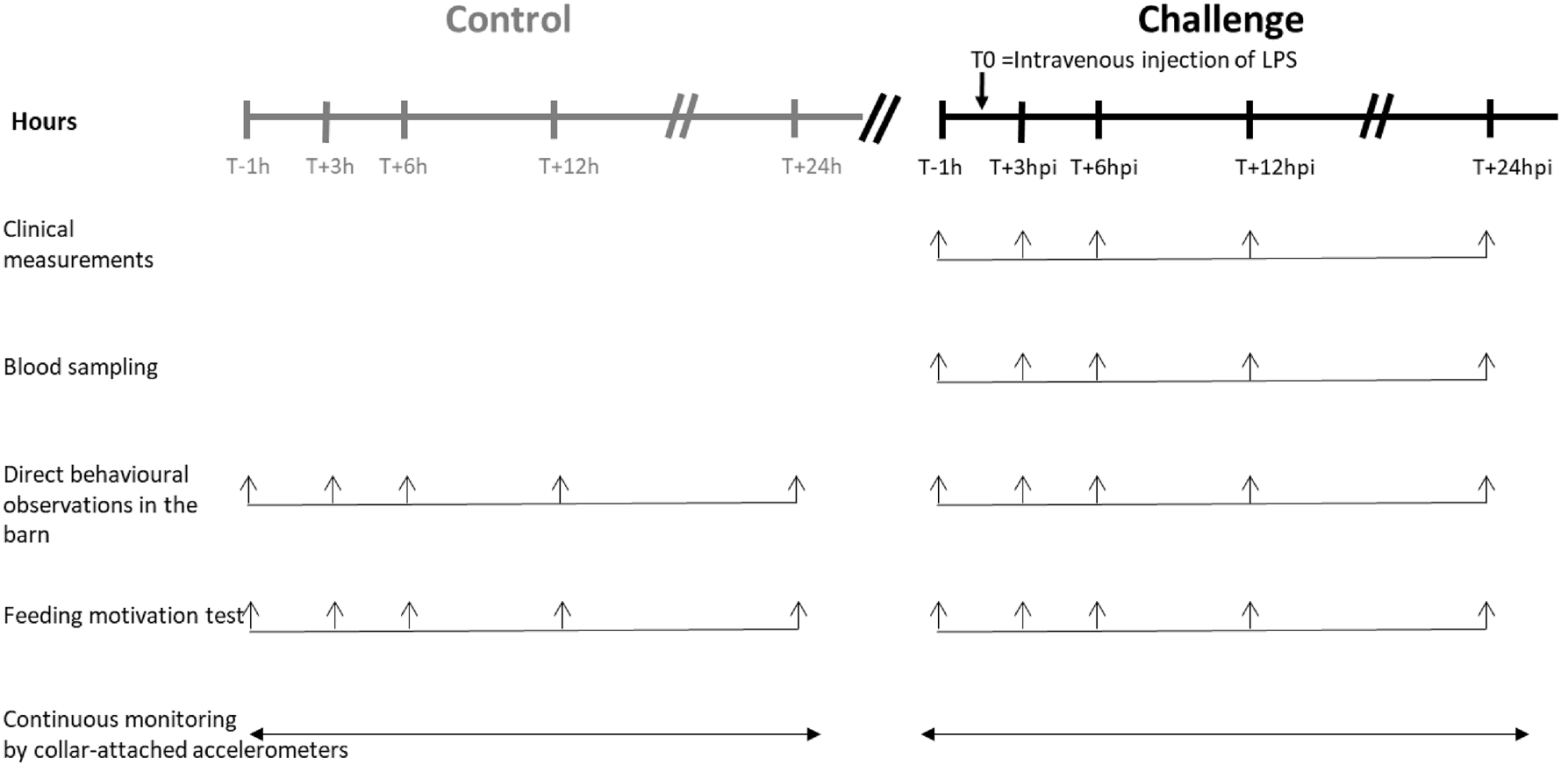
Schedule of measures to investigate the effects of intravenous injection (at T0) of lipopolysaccharide (LPS) (0.5 µg/kg of BW) on clinical and blood parameters and behavioural indicators in 14 Holstein cows. Each cow was first submitted to the control situation then challenged with LPS, or vice versa.

Behavioural observations, clinical examinations and blood sampling were performed at regular intervals from just before the LPS injection up to 24 h post-injection (hpi), by two experienced researchers or by continuously sensor monitoring of the cow’s activities and position. Five time-points were chosen to monitor the animal responses to the LPS challenge, based on the putative inflammatory response from acute phase to recovery^18,29,32^: T−1 h (just before the LPS challenge), at 3 hpi (T + 3 h) and 6 hpi (T + 6 h) to monitor the acute phase, then at 12 hpi (T + 12 h) and 24 hpi (T + 24 h). The clinical and blood data obtained at T−1 h served as baseline control (Fig. 1).

#### Direct behavioural observations in the barn and feeding motivation test

**Figure 2:**
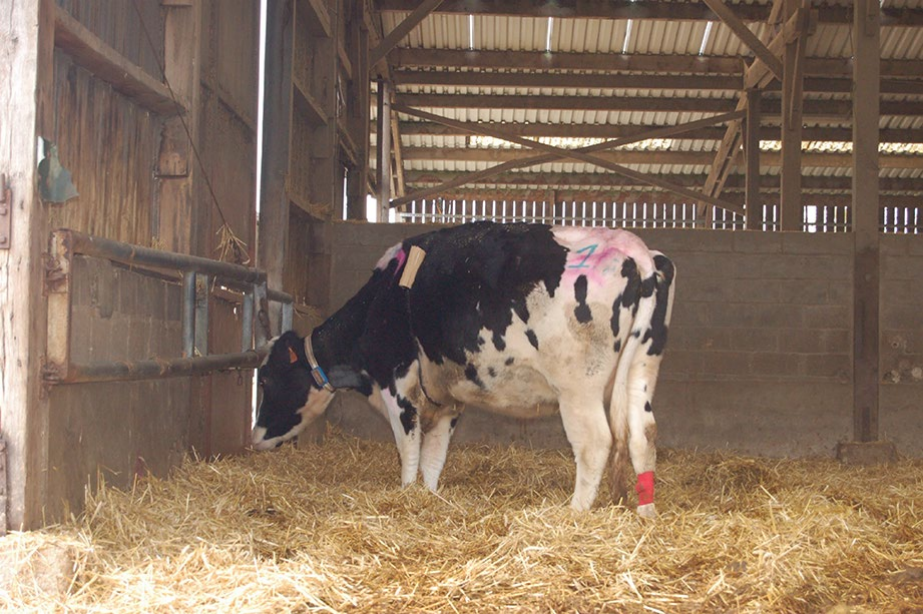
Cow displayed apathy, with head in down position and tail pressed her tail against vulva and udder.

**Figure 3:**
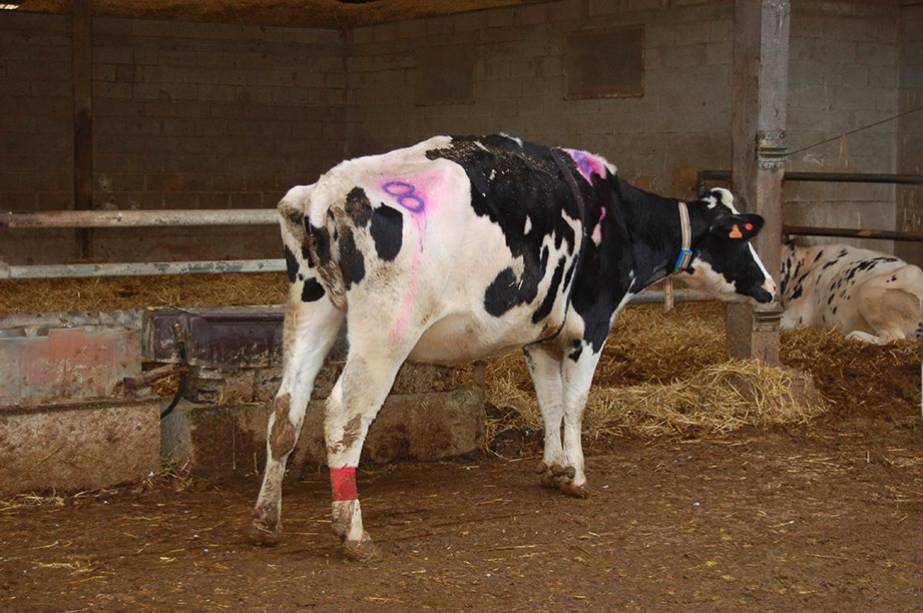
Cow displayed a hunched back, with hindlegs extended and tail pressed against vulva and udder.

**Figure 4:**
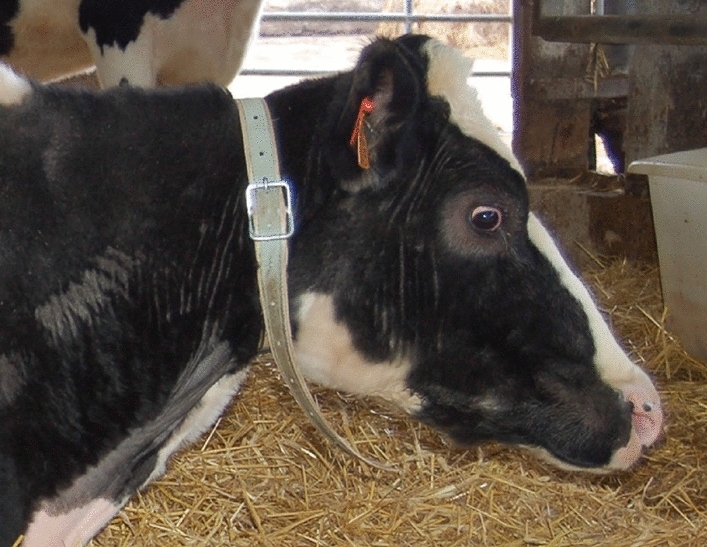
Cow displayed a wide-open position of the eyelids.

**Figure 5:**
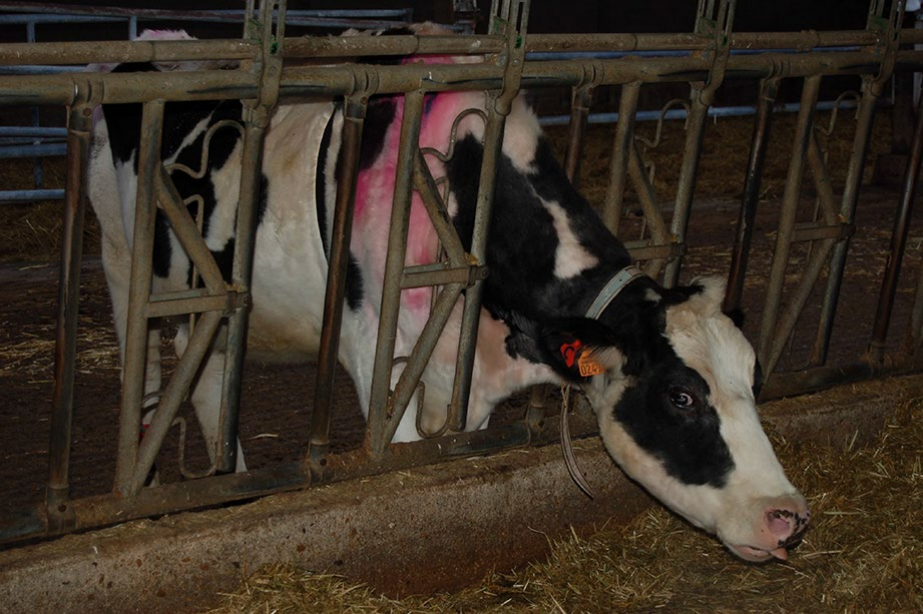
Cow displayed an orthopnoea posture: neck stretched and open mouth.

The cows’ general behaviour was recorded from 1 h before up to 24 h after the challenge, a few minutes before each five time-points, when the cows were free to move in their pen, before headlocked them. The observer was blind to the treatment applied and used instantaneous focal-animal sampling^33^. A few minutes before each time-point, she quietly approached the barn, observed the cow for 10–20 s approximatively, and completed an individual assessment of the cow’s behaviour by standing at the boundary of the barn, at 5–8 m from the focal cow so as not to disturb her. She was trained in observational analysis of animal behaviour, and checked absence or presence of 15 behavioural signs of sickness or pain selected from the literature, i.e. proximity to conspecifics, activity (resting, eating, or ruminating), apathy, posture (standing, balance, position of the head, back, legs, and tail), facial expression (position of ears, eyelids and nostrils), and miscellaneous clinical signs as seen at first glance. Definitions of the signs are given in Table 1 and partly illustrated in Figs. 2, 3, 4, 5.

**Table 1:** Description of the behaviour patterns detected by direct behavioural observations in the barn.

Direct behavioural observations	Description
Social proximity	Cow's distance to her first neighbour is less than 1 body length
Standing-up posture^11^	Cow is standing on all four legs
Resting^11^	Cow is possibly sleeping (eyes closed, head resting) or drowsy (eyes half-closed). The cow can be either standing up or lying down
Apathy^11^	Cow is not active, not sleeping and not ruminating, does not react to tactile, visual and/or audible environmental stimuli, or is orientated towards a wall
Feeding activity^34^	Cow is eating or ruminating
Unsteady balance^34^	Cow is standing or resting unsteadily, sometimes with body leaning against a wall; or standing with weight shifting on hind legs
Head down^34^	Cow’s head (poll) is below the line of the spinal column. The cow can be either standing up or lying down
Hunched back posture^35^	Cow is standing with its back arched
Extended forelegs position^35^	Cow is standing or lying with at least one foreleg partially or fully extended
Extended hindlegs position^35^	Cow is standing or lying with at least one hindleg partially or fully extended caudally
Pressed tail position^36^	Cow's central part of tail is pressed against vulva and udder and distal part of tail between hindlegs
Eyelids wide open^37^	Cow's pupil, iris and sclera are visible
Ears down^11,37^	Cow’s ears lower than spinal column, with increased gap between ears and the opening facing downwards
Strained nostrils^37^	Cow's nostrils sometimes dilated, possibly also with creases above the nostrils
Miscellaneous clinical signs	Cow displays dyspnoea and/or tremor and/or orthopnoea

A feeding motivation test was then carried out at each of the five time-points. The familiar caretaker placed a portion of the usual feed into the trough and called all of the cows as she does every day. The observer then noted, for each cow, whether it approached or not, and whether it ate the feed or not within 3 min after being called over by its familiar caretaker.

#### Physiological and clinical measurements, blood sampling and assays

Immediately after the behavioural observations and the feeding-motivation test, the cows were headlocked and an experienced veterinarian performed a clinical examination. The veterinarian recorded physiological parameters, i.e. rectal temperature (RT) with a thermometer, heart rate (HR) by counting heartbeats during a 20-s period with a stethoscope, rumen motility rate (RMR) for 2 min with a stethoscope, respiratory rate (RR) by counting the number of expansions of the thoracic wall during a 30-s period. The veterinarian noted whether the cow displayed signs of dyspnoea, signs of tremor, and other miscellaneous signs, and then collected blood samples into Na2-EDTA-coated vacutainer tubes by puncture of the coccygeal vein. The cow was then released from the headlock.

The blood samples were centrifuged at 3000 g for 20 min at 4 °C, and the plasma was collected and frozen at −20 °C to determine cortisol, pro-inflammatory cytokines IL-1β, IL-6, and TNF-α, and the haptoglobin inflammatory parameter. Plasma cortisol concentration was determined by enzyme assay^38^. Concentrations of pro-inflammatory cytokines were determined using a custom bovine MilliPlex xMAP cytokine assay (Merck Millipore, France)^39^. Data was recorded on a MagPix flow cytometer using Xponent software (Luminex, Austin, TX). Plasma haptoglobin concentration was determined by immunoprecipitation^40^. All plasma determinations were performed at all the defined time-points from T−1 h to T + 24 hpi except haptoglobin that was only determined at T−1 h, T + 12 hpi and T + 24 hpi.

#### Continuous monitoring of cow activities and position by collar-attached accelerometers

The data from the accelerometers was processed using the FARMLIFE software (ITK/NewMedria, Chateaubourg, France) to provide information on animal activity and position. The accelerometer measures changes of inclination, and lateral and vertical accelerations, from which the behaviour and the posture of the animals are derived by the ITK/NewMedria Company. Every 5 min, the cow was classified as ingesting feed or ruminating or showing no motion (without activity) or in another activity according to the state in which it had spent most of it time over the previous five minutes. The position of the cow, i.e. standing up *vs*. lying down, was also recorded. The sensors are able to record eating and rumination time with a precision of 89–90%^41^ and from 83% for the position to 90% for no activity^42^. This commercial device was recently used in bovine behaviour study^43^. We then calculated, for each cow, the estimated proportion of time spent per hour in each activity and in each position.

### Statistical analyses

For the cows in the CHALLENGE situation, we modelled time-course changes in cortisol, cytokine and haptoglobin levels, RT, HR, RMR and RR using linear mixed-effects models with time (T + 3 hpi, T + 6 hpi, T + 12 hpi, T + 24 hpi *vs.* T−1 h) for cortisol, cytokines, RT, HR, RMR and RR and with T + 12 hpi, T + 24 hpi *vs*. T−1 h for haptoglobin, with age (old *vs.* young cows) and their interaction (age x Time) as fixed effects and ‘cow’ as random effect. The T−1 h time-point was time before LPS injection (i.e. the control level). Cortisol, IL-6, and TNF-α concentrations were log-transformed to satisfy conditions of normal distribution and homogeneity of residuals.

Behavioural data recorded through direct observations in the barn and the feeding motivation test were analyzed as qualitative paired series using McNemar tests. CHALLENGE *vs*. CONTROL and old *vs*. young cows were compared at each timepoint (T−1 h, T + 3 hpi, T + 6 hpi, T + 12 hpi, T + 24 hpi).

For the time spent in each activity (ingesting, ruminating, without activity) and in the ‘standing up’ position recorded by collar-attached accelerometers, we conducted daily (24 h) then hourly analyses. First, we used a linear mixed effect model with situation (CHALLENGE *vs.* CONTROL), age (old *vs*. young cow) and their interaction (age x situation) as fixed factors and ‘cow’ as random effect. Second, we analyzed the time spent in each activity and position using a linear mixed-effects model with Time every hour from 0 to 24 h, situation, age, and Time × situation × age interaction as fixed effects, and ‘cow’ as random effect.

Taking into account all behaviours recorded by direct observations, the feeding motivation test and continuous monitoring of cow activities and position by the collar-attached accelerometers, we conducted factorial discriminant analysis to differentiate cows according to age (young or old), situation (CONTROL or CHALLENGE), and the five timepoints (T−1 h, T + 3 hpi, T + 6 hpi, T + 12 hpi, T + 24 hpi). Discriminant analysis identifies linear combinations of variables that best discriminate groups. The analysis was run on the behavioural data that had showed significant changes in univariate analyses (McNemar test; see above) and on the data from the continuous monitoring of cow activities and position, which was limited to the three hours before each timepoint in order to match it to data from direct observations.

All analyses were performed using R software version 4.0.5^44^. The *lmer* function from the *lme4* package was used for the linear mixed effect models^45^ and the *emmeans* package was used to calculate least square means. The threshold for significance of a fixed effect was set at a t-value ≥|2| and a Tukey-adjusted *p* value ≤ 0.05^46^. The normal distribution and homogeneity of residuals were visually verified using plots of residuals and quantile–quantile plots of residuals and random effects. For the McNemar test, a difference was considered significant at *p* ≤ 0.05 and as a trend when at 0.05 < *p* ≤ 0.10. The *ade4* package was used to perform the factorial discriminant analysis. To interpret the discriminant components of the factorial discriminant analysis, we focused on variables with a correlation of absolute value with the axis >|0.4|. Only significant results and tendencies are reported in the "Results" section. Results from direct behavioural observations in the barn and the feeding motivation test are reported as number of cows. The results of clinical measurements, analyses of blood samples, and continuous monitoring of cow activities and position are reported as least square means ± Standard Error (SE).

## Results

### Cows reaching an endpoint and final dataset

Two cows reached endpoints. One young cow suffered a hip dislocation between the CONTROL and CHALLENGE situations and was therefore euthanized; all data from this cow were excluded from the dataset. One old cow displayed downer cow syndrome at 24 hpi, with sternal recumbency, apathy, subnormal RT (37.7 °C), ruminal atony and anorexia. It was given an oral ruminatoric administered (TRANSITONYL, Ceva Santé Animale, Libourne, France) added to water and pumped into the rumen, and it lifted up after 48 h and achieved totally recovery after 15 days. The data from this cow were kept in the final dataset. One young cow was excluded from the dataset due to no sensor data records in the CONTROL situation. The final dataset therefore contained 12 cows: 6 old cows and 6 young cows.

### Clinical, physiological and stress responses to the inflammatory challenge

**Figure 6:**
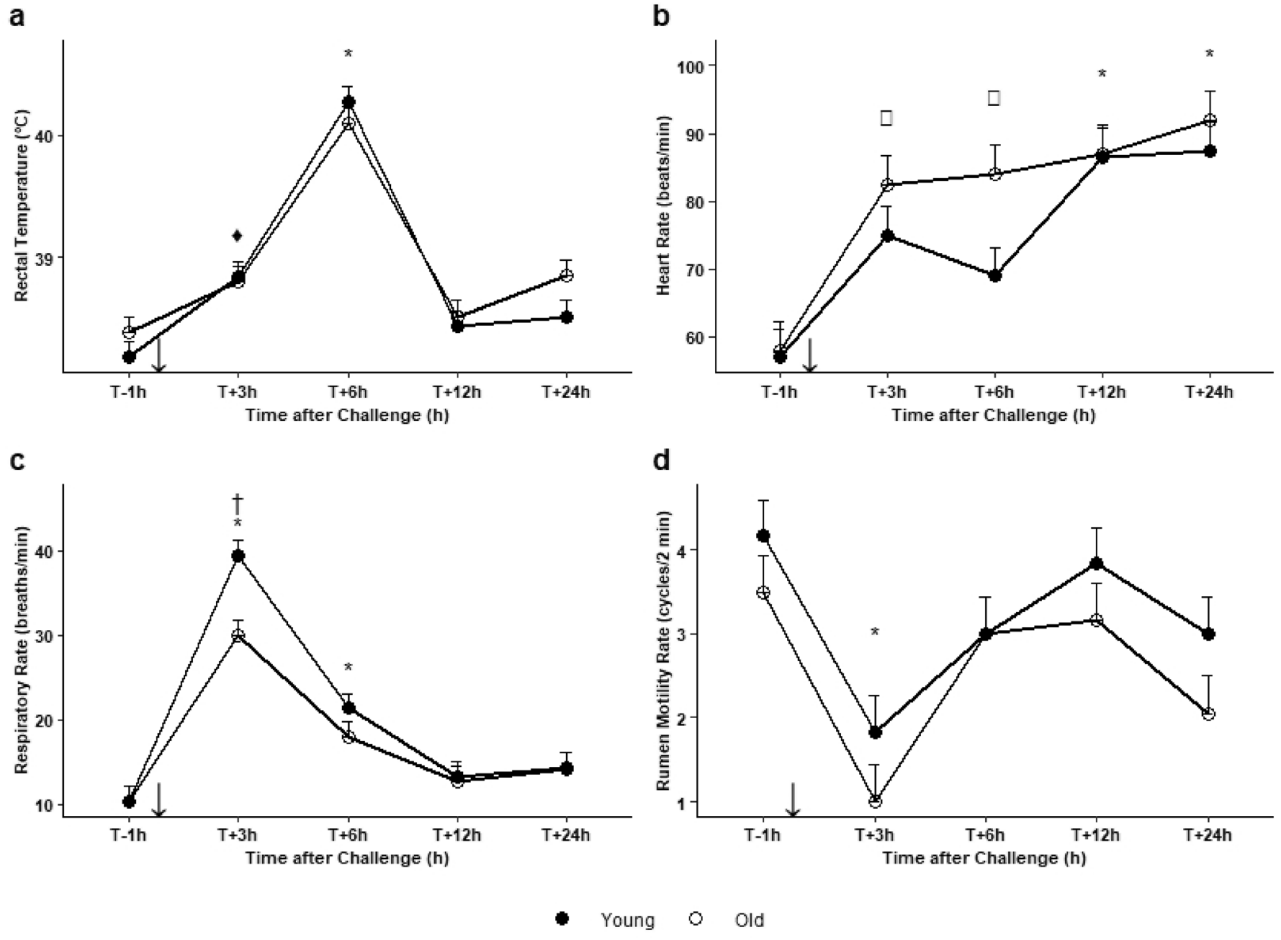
Changes in (**a**) rectal temperature, (**b**) heart rate, (**c**) respiratory rate, and (**d**) rumen motility rate in 6 old (white dots) and 6 young (dark dots) Holstein cows one hour before (T−1 h) and at 3, 6, 12 and 24 h after intravenous injection of LPS (0.5 µg/kg of BW) (black arrow). Data are presented as least squares means (± SE). Means flagged with  * indicate significant differences between the values obtained at a given time-point and baseline value obtained at T−1 h regardless of age, with ^♦^ for young cows only and □ for old cows only (*p* ≤ 0.05). Means flagged † indicate significant difference between old and young cows at a specific time-point (*p* ≤ 0.05).

The cows responded clinically to the LPS bolus injection, seen from the comparison of values at T-1 h with those obtained later on (Fig. 6, plus detailed output of the models in Supplementary Table S1). The four parameters analyzed (RT, HR, RR and RMR) showed similar time-course patterns for both young and old groups. Nevertheless, some specific features emerged. In young cows, compared to T−1 h (38.2 ± 0.1 °C), RT significantly increased at T + 3 hpi to reach 38.8 ± 0.1 (*P* = 0.02). In both groups, compared to T−1 h (young cows: 38.2 ± 0.1 °C; old cows: 38.4 ± 0.1 °C), RT significantly increased at T + 6 hpi to reach 40.3 ± 0.1 °C in young cows and 40.1 ± 0.1 °C in old cows (*P* < 0.0001). Then, RT progressively fell back down to baseline values. In old cows, compared to T−1 h (58 ± 4.2 beats/min), HR increased significantly from T + 3 hpi (82.5 ± 4.2 beats/min, *P* = 0.007) and continued to increase up to T + 24 hpi (92 ± 4.2 beats/min). In young cows, compared to T−1 h (57 ± 4.2 beats/min), HR increased significantly and reached a plateau until the end of the experimental period (86.5 ± 4.2 at T + 12 hpi, *P* = 0.0006, 87.5 ± 4.2 beats/min at T + 24 hpi, *P* = 0.0003). In both groups, compared to T−1 h (young cows: 10.3 ± 1.8 breaths/min; old cows: 10.3 ± 1.8 breaths/min), RR increased significantly to peak at T + 3 hpi (39.3 ± 1.8 breaths/min, *P* < 0.0001 for young cows, 30.0 ± 1.8 breaths/min, *P* < 0.0001 for old cows). At T + 3 hpi, RR was lower in the old cows than young cows (*P* = 0.02). Compared to T−1 h (young cows: 4.2 ± 0.4 contractions/2 min; old cows: 3.5 ± 0.4 contractions/2 min), RMR decreased significantly at T + 3 hpi and similarly in both groups of cows (1.8 ± 0.4 contractions/2 min, *P* = 0.003 for young cows and 1.0 ± 0.4 contractions/2 min, *P* = 0.001 for old cows). The values returned to baseline from T + 12 hpi. Miscellaneous clinical signs (dyspnoea and tremor) were noted in 7 cows at T + 6 hpi (Table 2; *p* = 0.02, McNemar test), none of which had shown miscellaneous clinical signs at T−1 h.

**Table 2:**
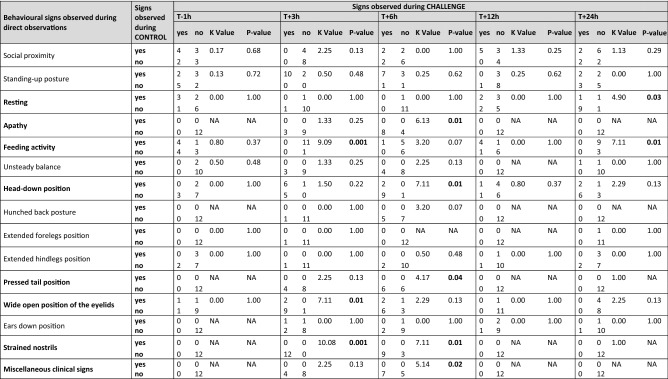
Concordance of behavioural signs in 12 Holstein cows under LPS CHALLENGE and CONTROL situations at T−1 h, T + 3 h, T + 6 h, T + 12 h and T + 24 h after intravenous injection of LPS (0.5 µg/kg of BW) (or equivalent time in the CONTROL situation).

McNemar tests served to check whether cows had changed their behaviour between the two situations (change exists when *p* ≤ 0.05). For instance, regarding apathy at T + 6 h, 8 cows that were not apathetic in the CONTROL situation became apathetic in the CHALLENGE situation, while the 4 other cows were not apathetic in both the CHALLENGE and CONTROL situations (*p* = 0.01). NA corresponds to no cows changing their behaviour between the two situations.

Significant values are in bold.

**Figure 7:**
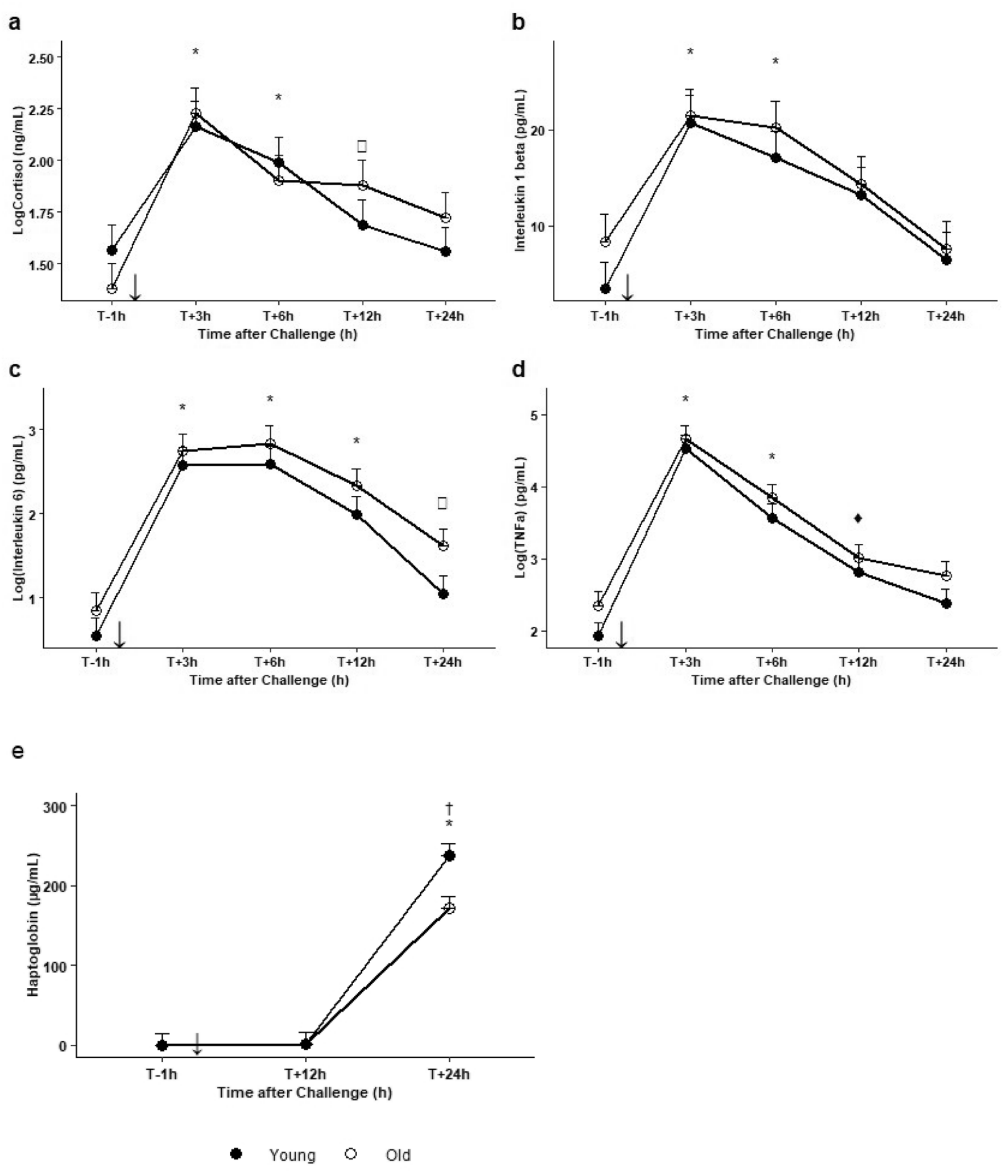
Changes in plasma concentration of (**a**) cortisol, (**b**) IL-1β, (**c**) IL-6, (**d**) TNF-α and (**e**) haptoglobin in 6 old (white dots) and 6 young (dark dots) Holstein cows one hour before (T−1 h) and at 3 h, 6 h, 12 h and 24 h after intravenous injection of LPS (0.5 µg/kg of BW) (black arrow). Data are presented as least squares means (± SE). Means flagged with * indicate significant differences between the values obtained at a given time-point and baseline value obtained at T−1 h regardless of age, with ^♦^ for young cows only and □ for old cows only (*p* ≤ 0.05). Means flagged † indicate significant difference between old and young cows at a specific time-point (*p* ≤ 0.05).

All blood parameters changed significantly (Fig. 7 plus detailed output of the models in Supplementary Table S2). Compared to T−1 h (young cows: 37.2 ± 1.3 ng/mL; old cows: 24.0 ± 1.3 ng/mL), plasma cortisol concentration increased significantly and peaked at T + 3 hpi (144.5 ± 1.3 , *P* = 0.0003 for young cows, 169.8 ± 1.3, *P* < 0.0001 for old cows) and fell back to initial baseline level by T + 12 hpi for young cows and T + 24 hpi for old cows. There was a similar significant elevation to peak at T + 3 hpi compared to T−1 h for plasma IL1-β (20.7 ± 2.8 *vs.* 3.5 ± 2.8 pg/mL, *P* = 0.0001 for young cows and 21.5 ± 2.8 *vs.* 8.4 ± 2.8 pg/mL, *P* = 0.005 for old cows), IL-6 (377.6 ± 1.6 *vs.* 3.5 ± 1.6 pg/mL, *P* < 0.0001 for young cows and 548.3 ± 1.6 *vs.* 7.0 ± 1.6 pg/mL, *P* < 0.0001 for old cows), and TNF-α (33 884.4 ± 1.5 *vs.* 85.1 ± 1.5 pg/mL, *P* < 0.0001 for young cows and 44 668.4 ± 1.5 *vs.* 223.9 ± 1.5 pg/mL, *P* < 0.0001 for old cows) and the values had returned to baseline at T + 24 hpi except for IL-6 in old cows which remained significantly higher than at T−1 h (40.6 ± 1.6 pg/mL, *P* = 0.03). There were no significant between-age differences in plasma cortisol and cytokine concentrations. Plasma haptoglobin concentration was increased at T + 24 hpi in both young cows (237.9 ± 14.9 *vs.* 0.0 ± 14.9 mg/mL, *P* < 0.0001) and old cows (172.1 ± 14.9 mg/mL *vs.* 0.0 ± 14.9 mg/mL, *P* < 0.0001) but the increase was significantly less marked in old cows than young cows (*P* = 0.04).

### Cow behaviour from direct observations in the barn and feeding motivation test

The number of cows expressing each modality of behaviour did not differ between old and young cows. At T−1 h and T + 12 hpi, there were no significant differences between the CONTROL *vs*. CHALLENGE situations (McNemar test; *P* > 0.05) (Table 2).

At T + 3 hpi, there were significant differences in behaviour between CONTROL and CHALLENGE situations in both cow age groups. Eleven cows stopped feeding in the CHALLENGE situation whereas they fed in the CONTROL situation. Nine cows had their eyelids wide open with pupil, iris and sclera visible in the CHALLENGE situation whereas only their pupil and iris were visible in the CONTROL situation (Fig. 4). Twelve cows had strained nostrils in the CHALLENGE situation whereas none of them had strained nostrils in the CONTROL situation.

At T + 6 hpi, there were further differences in behaviour between CONTROL and CHALLENGE situations in both cow age groups. Similar to T + 3 h in both cow age groups, feeding behaviour and position of nostrils differed between the two situations. Five cows stopped feeding in the CHALLENGE situation whereas they fed in the CONTROL situation, and 9 cows in the CHALLENGE situation whereas none of them had strained nostrils in the CONTROL situation. Other behaviours were modified: in the CHALLENGE situation, 8 cows became apathetic, 9 cows carried their head down, 5 cows stood with their back hunched, 6 cows pressed their tail against their udder, and 7 cows displayed clinical signs (dyspnoea, orthopnoea or tremor) whereas none of them displayed such behaviours in the CONTROL situation (Figs. 2, 3, 5).

At T + 24 hpi, most of these differences were no longer observed, although depressed feeding behaviour was still visible, and 9 cows were resting whereas they were not in the CONTROL situation.

When tested for feeding motivation, 12 and 11 cows came and fed in the CONTROL situation at T + 3 hpi and T + 6 hpi, respectively, but none did so in the CHALLENGE situation (*P* = 0.001 and 0.003). There were no differences between CHALLENGE and CONTROL in both cow age groups at T−1 h (11 cows came and fed in the CONTROL and CHALLENGE situations), T + 12 hpi (6 cows came and fed in the CONTROL and CHALLENGE situations) and T + 24 hpi (7 cows came and fed in the CONTROL and CHALLENGE situations) (*P* = 1, 0.22 and 0.13, respectively).

### Cow activities and position monitored by collar-attached accelerometers

As the results of daily activity analysis, over the 24-h period studied (1440 min), cows in the CONTROL situation spent 415.0 ± 16.5 min per day ingesting, 496.0 ± 22.5 min ruminating, 338.0 ± 23.9 without activity, and the rest of the time in another activity. They spent 842.0 ± 45.9 min standing up (as opposed to lying). In the CHALLENGE situation, compared to the CONTROL situation, they spent significantly less time ingesting (−310.0 ± 16.5 min, *P* < 0.0001) and ruminating (−44.0 ± 22.5 min, *P* < 0.0001) and significantly more time without activity (+ 560.0 ± 23.9 min, *P* < 0.0001) and standing up (+ 226.0 ± 45.9 min, *P* = 0.003). In the CHALLENGE situation, old cows spent significantly more time without activity than young cows (+ 195.0 ± 32.9 min, *P* = 0.003) whereas there was no between-age difference in the CONTROL situation (Table 3).

**Table 3:** Least squares (Ls) means in minutes ± Standard Error (SE) and Tukey-adjusted p-values from linear mixed-effect models of time spent ingesting (min), time spent ruminating, time spent without activity, and time spent standing up over 24 h (daily analysis) stratified by situation (CHALLENGE vs. CONTROL), and interaction between age (old *vs*. young) and situation, in 6 old and 6 young Holstein cows intravenously injected with LPS (0.5 µg/kg of BW) at T0.

Parameters	Fixed and random factors	Ls means ± SE	t-value	*p* value
Time spent ingesting (min per 24 h)	Simple fixed factors			
	Control	415.0 ± 16.5		
	Challenge	105.0 ± 16.5	13.3	< 0.0001
	Interacting fixed factors			
	Old control	431.7 ± 23.3		
	Young control	398.3 ± 23.3	1	0.74
	Old challenge	82.5 ± 23.3		
	Young challenge	127.5 ± 23.3	1.3	0.53
	Random effects	Variance ± SD		
	Animal	0.0	–	–
	Residuals	3250 ± 57.01	–	–
Time spent ruminating (min per 24 h)	Simple fixed factors			
	Control	496.0 ± 22.5		
	Challenge	252.0 ± 22.5	10.9	< 0.0001
	Interacting fixed factors			
	Old control	513.0 ± 31.8		
	Young control	478.0 ± 31.8	0.8	0.86
	Old challenge	202.0 ± 31.8		
	Young challenge	301.0 ± 31.8	2.2	0.17
	Random effects	Variance ± SD		
	Animal	3083 ± 55.5	–	–
	Residuals	2980 ± 54.6	–	–
Time spent without activity (min per 24 h)	Simple fixed factors			
	Control	338.0 ± 23.9		
	Challenge	898.0 ± 23.9	17.4	< 0.0001
	Interacting fixed factors			
	Old control	338.0 ± 33.8		
	Young control	338.0 ± 33.8	0.02	1
	Old challenge	995.0 ± 33.8		
	Young challenge	800.0 ± 33.8	4.1	0.003
	Random effects	Variance ± SD		
	Animal	612.5 ± 24.8	–	–
	Residuals	6231.0 ± 78.9	–	–
Time spent standing up (min per 24 h)	Simple fixed factors			
	Control	842.0 ± 45.9		
	Challenge	1068.0 ± 45.9	3.89	0.003
	Interacting fixed factors			
	Old control	870.0 ± 65.0		
	Young control	815.0 ± 65.0	0.6	0.93
	Old challenge	1148.0 ± 65.0		
	Young challenge	989.0 ± 65.0	1.7	0.34
	Random effects	Variance ± SD		
	Animal	5092 ± 71.4	–	–
	Residuals	20,225 ± 142.2	–	–

**Figure 8:**
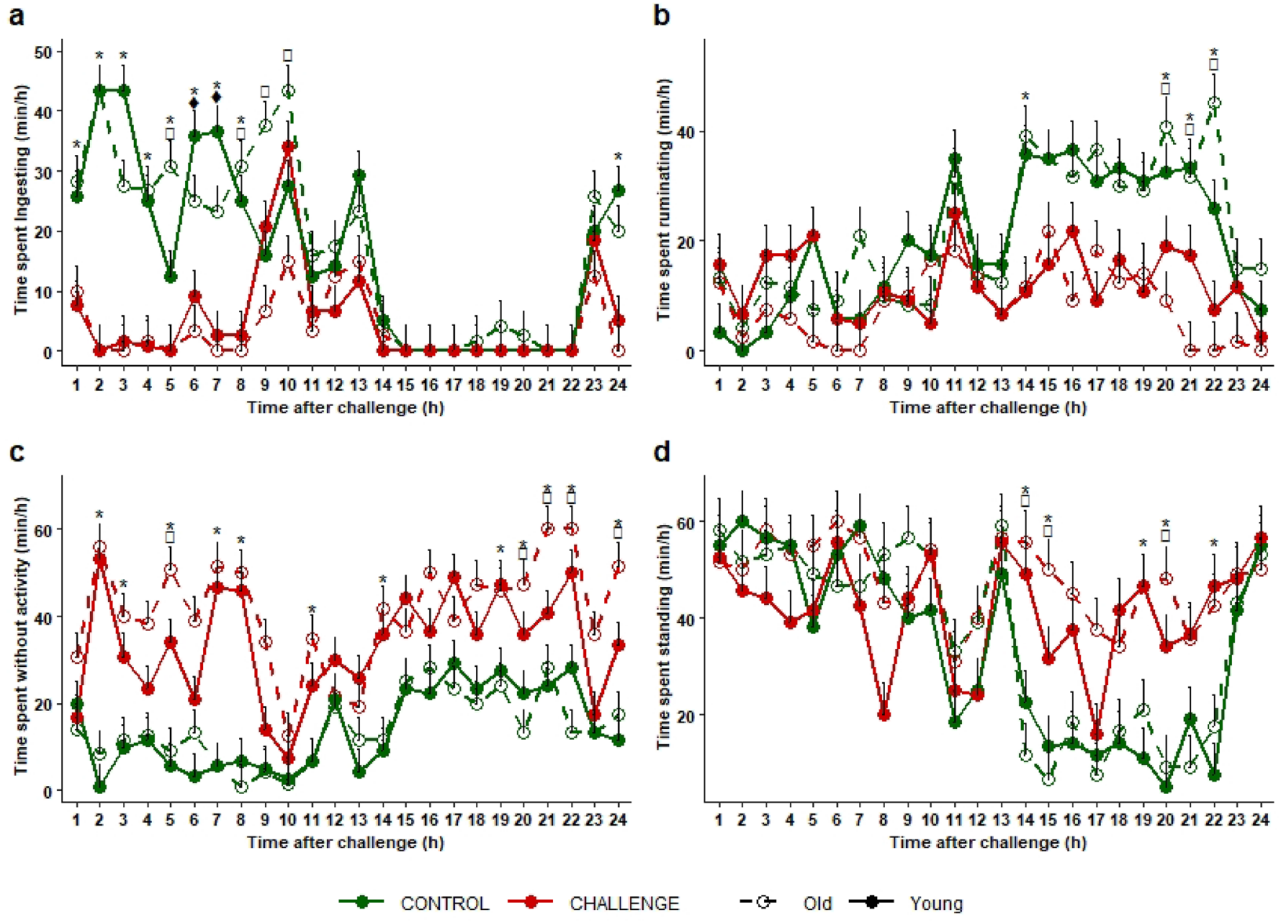
Changes in time spent (**a**) ingesting, (**b**) ruminating, (**c**) without activity, and (**d**) standing up in minutes per hour in 6 old (white dots and hatched lines) and 6 young (filled dots and solid lines) Holstein cows during 24 h (hourly analysis) before intravenous injection of LPS (T0; 9:30 am) (0.5 µg/kg of BW) (CONTROL, in green) and after (CHALLENGE, in red). Data are presented as least squares means (± SE). Means with * indicate significant differences between timepoints and situations (CHALLENGE vs. CONTROL) regardless of age, with ^♦^ for young cows only, and □ for old cows only (*p* ≤ 0.05).

Hourly activity analysis showed that right after the LPS injection, the cows spent less time ingesting between hour 1 to hour 8 for all cows and until hour 10 for old cows than in the CONTROL situation (ingesting by old cows: between 0 and 15.0 ± 4.2 min/h in the CHALLENGE situation *vs.* between 27.5 and 43.3 ± 4.2 min/h in the CONTROL situation, *P* ≤ 0.02; ingesting by young cows: between 0 and 19.2 ± 4.2 min/h in the CHALLENGE situation *vs.* between 33.7 and 43.3 ± 4.2 min/h in the CONTROL situation, *P* ≤ 0.03). The cows also spent more time without activity between hour 2 to hour 8 than in the CONTROL situation (not activity by old cows: between 50 and 55.8 ± 5.2 min/h in the CHALLENGE situation *vs.* between 0.8 and 9.2 ± 5.2 min/h in the CONTROL situation, *P* ≤ 0.0001; no activity by young cows: between 45.8 and 53.3 ± 5.2 min/h in the CHALLENGE situation *vs.* between 0.8 and 6.7 ± 5.2 min/h in the CONTROL situation *P* ≤ 0.0002) (Fig. 8a and c, plus detailed output of the models in Supplementary Table S3).

Towards the end of the 24 hpi period (from hour 19), time spent without activity was again increased in the CHALLENGE situation compared to CONTROL, especially for the older cows (between 47.5 and 60.0 ± 5.2 min/h in the CHALLENGE situation *vs.* between 13.3 and 28.3 ± 5.2 min/h in the CONTROL situation from hour 19 to 24 for old cows, *P* ≤ 0.05). At hour 24, time spent ingesting was again reduced in the CHALLENGE situation compared to CONTROL (2.5 vs. 23.3 ± 3.0 min/h, *P* < 0.001) (Fig. 8a and c, plus detailed output of the models in Supplementary Table S3).

Time spent ruminating and standing up remained stable along the 24 h in the CHALLENGE situation in both old and young cows. In contrast, in the CONTROL situation, from hour 14 to hour 22 (during the night), time spent ruminating increased and time spent standing up decreased, resulting in significant differences to the CHALLENGE situation (ruminating: between 3.7 and 11.5 ± 3.7 min/h in the CHALLENGE situation *vs.* between 35.4 and 37.5 ± 3.7 min/h in the CONTROL situation, *P* < 0.02; standing up: between 44.6 and 52.6 ± 4.6 min/h in the CHALLENGE situation *vs.* between 12.5 and 17.1 ± 4.6 min/h in the CONTROL situation *P* < 0.0009). At the end of the CHALLENGE, old cows spent significantly less time ruminating than young cows (Fig. 8b and d).

### Discrimination of cow behavioural responses between situations, ages, and time-points

**Figure 9:**
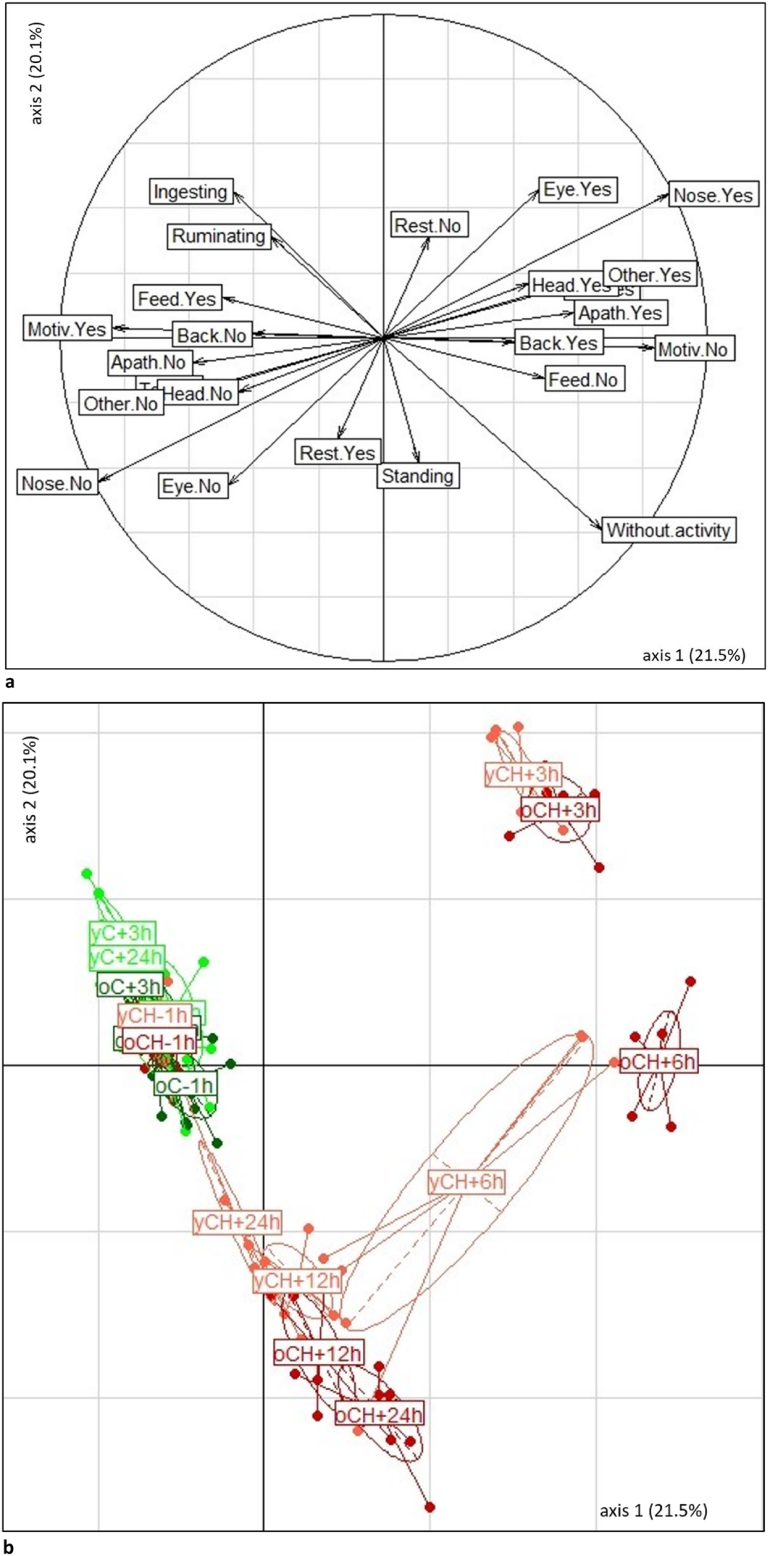
Factorial map of the discriminant analysis on behavioural signs in 6 old and 6 young Holstein cows under LPS CHALLENGE and CONTROL situation at T−1 h, T + 3 h, T + 6 h, T + 12 h and T + 24 h after intravenous injection of LPS (0.5 µg/kg of BW) (or equivalent time in the CONTROL situation): loadings of (**a**) the variables and (**b**) the individuals on axis 1 and axis 2 of the factorial discriminant analysis. Due to plot size limitations, the label tags have been simplified on the plot: “oCH + 3 h” corresponds to old cows in the CHALLENGE situation at 3 h after injection, “yC + 3 h” corresponds to young cows in the CONTROL situation at 3 h after T0, and so on. See Table 4 for details of the names of the variables and all variable-to component correlations.

The discriminant analysis was run on ten binomial behavioural variables and four quantitative continuous activities or positions. Components 1 and 2 of the discriminant analysis accounted for 21.5 and 20.1% of the total variance, respectively. Component 1 captured a gradient of sickness and pain: it opposed strong sickness and pain reactions (positive correlations to component) *vs.* no sickness and pain reactions (negative correlations to component) characterized by apathy, time spent without activity, back–tail–head postures, facial expressions (eyelids, nostrils), and low feeding (motivation, activity and time spent ingesting). Component 2 captured a gradient of arousal: it opposed a low level of arousal (negative correlations to component) vs. high level of arousal (positive correlations to component) characterized by high time spent ingesting, low time spent without activity, eyes wide open and strained nostrils (Table 4 and Fig. 9a). These two components thus discriminated five groups of cows (Fig. 9):

- A group with all cows in the CONTROL situation and cows 1 h before the LPS injection that had negative values on Component 1 and neutral values on Component 2, i.e. no sickness or pain responses and neutral arousal.
- Cows at 3 hpi—whatever their age—that had positive values on Components 1 and 2, i.e. strong sickness and pain responses and a high level of arousal.
- Old cows at 6 hpi with very high values on Component 1 and neutral values on Component 2, i.e. cows with very strong sickness and pain responses but neutral arousal.
- Young cows at 6 hpi that had moderately positive values on Component 1 and moderately negative values on Component 2, i.e. moderate sickness and pain responses and a moderately low level of arousal.
- Cows at 12 hpi and 24 hpi that had neutral values on Component 1 and negative values on Component 2, i.e. low pain and sickness responses and low arousal. Two subgroups were be distinguished according to age: very low arousal in old cows and low arousal in young cows.

**Table 4:** Factorial discriminant analysis on behavioural signs in 6 old and 6 young Holstein cows under LPS CHALLENGE and CONTROL situations at T−1 h, T + 3 h, T + 6 h, T + 12 h and T + 24 h after intravenous injection of LPS (0.5 µg/kg of BW) (or equivalent time in the CONTROL situation): proportion of variance explained by each component, and variable-to-component correlations.

Factorial discriminant analysis		Component 1	Component 2
Proportion of total variance explained by component (%)		21.50	20.10

Contribution of each variable to component			
Variables	Abbreviations		
Strained nostrils: Yes	Nose.Yes	0.88	0.45
Motivated to feed: No	Motiv.No	0.84	−0.03
Dyspnea and/or tremor: Yes	Other.Yes	0.68	0.20
Time spent without moving	Without moving	0.67	−0.59
Apathy: Yes	Apath.Yes	0.59	0.08
Pressed tail: Yes	Tail.Yes	0.56	0.16
Feeding activity: No	Feed.No	0.50	−0.13
Eyelids wide open: Yes	Eye.Yes	0.48	0.46
Head down: Yes	Head.Yes	0.45	0.17
Hunched back: Yes	Back.Yes	0.40	−0.01
Resting: No	Rest.No	0.14	0.31
Time spent standing	Standing	0.11	−0.39
Resting: Yes	Rest.Yes	−0.14	−0.31
Time spent ruminating	Ruminating	−0.35	0.31
Hunched back: No	Back.No	−0.40	0.01
Head down: No	Head.No	−0.45	−0.17
Time spent ingesting	Ingesting	−0.47	0.45
Eyelids wide open: No	Eye.No	−0.48	−0.46
Feeding activity: Yes	Feed.Yes	−0.50	0.13
Pressed tail: No	Tail.No	−0.56	−0.16
Apathy: No	Apath.No	−0.59	−0.08
Dyspnoea and/or tremor: No	Other.No	−0.68	−0.20
Motivated to feed: Yes	Motiv.Yes	−0.84	0.03
Strained nostrils: No	Nose.No	−0.88	−0.45

Based on these distinctions, young cows appeared to have recovered quicker than old cows, with less marked responses at 6, 12, and 24 hpi.

## Discussion

The 12 cloned Holstein cows submitted to a systemic inflammatory challenge through intravenous bolus injection of LPS displayed subtle signs of sickness and pain behaviours (apathy, changes of facial expression and body postures, reduction in feeding motivation and ingestion). These signs developed rapidly (within 3 hpi) and lasted for at least 3 h. They were detected by direct visual observations whatever the age of the cows. Collar-attached accelerometers mounted on the animals were able to detect changes in basic activities and position earlier and for a longer period than direct observations. Cows spent less time ingesting during the day in the first hour after LPS injection and for 8 or 10 h after (depending on age), then again at 24 hpi which corresponded to the end of the monitoring period. They spent more time without activity from 2 hpi through to 24 hpi. They also spent less time ruminating and more time standing up during the night, i.e. from 14 to 22 hpi. The changes in time spent ruminating and without activity were more marked in the older cows than in the younger cows, especially at the end of the monitoring period. Behaviours recorded by direct observations and by continuous monitoring of cow activities via collar-attached accelerometers were able to identify the sickness and pain-related behaviour signs in the cows after onset of inflammation and for the following 24 h. These behavioural responses were more marked in the older cows.

The cows included in our study were derived by cloning a single genotype by somatic nuclear transfer^30,31^. Many studies have shown that cloned cows can be considered physiologically very close to normal but not totally normal^47^. Indeed, until two months of age, the body temperature and plasma leptin concentration were higher and thyroid hormone (T4) levels were lower in cloned calves than in noncloned ones^48^, suggesting variations in metabolism. Other cloned heifers monitored until 14 months of age showed differences in concentrations of growth factors compared to noncloned counterparts, but the differences remained within normal ranges^49^, suggesting that variations in metabolism between clones and non-clones were a result of a differences in genetic merit, since the clones were produced from the somatic cells of a single animal. Note too that the cloned cows used in this study are older than dairy cows bred commercially in France (five years on average in France^50^) and not had a career as producers: the young cows group had only been through a single pregnancy/lactation. The old cows group were only used to produce oocytes to be used for in vitro fertilization. These differences in career between the cows in this study and commercial dairy cows are liable to preserve the animal’s metabolism, and so these animals were not, therefore, entirely representative of Holstein cows raised on commercial dairy farms. However, in terms of behavioural responses, cloned cows would be not very different from noncloned cows reared on commercial farms^51^. Indeed, a group of cloned heifers observed in stable groups or in isolation showed no difference in social behaviour compared to heifers born from artificial insemination. Only exploratory behaviour was more marked in cloned heifers. These cloned heifers had benefited from more attentive care during the postnatal period and had more interactions with humans than controls. This care difference in exposure to care could have favoured their exploration performances^51^. The sickness and pain-related behaviours detected in the 12 cloned cows used here would thus not depend on the fact that the cows were clones.

Physiological markers of inflammation and behavioural responses to stress and pain vary with genotype. In genetically stable heifers (selected over 50 years to be genetically homogeneous) subjected to the same LPS challenge as the one used here, the difference in pro-inflammatory (TNF-α, IL-6), inflammatory (SAA) and rectal temperature responses compared to their contemporary heifers were explained by genetic alterations^25^. Genetic factors have an influence on behavioural responses in animals such as fear and fright responses in ruminants^15^ and Japanese quail^60^. In rodents, the behavioural response to pain is more variable between two strains than within the same strain where all individuals share identical loci^61^. Here, working with clones allowed us to study behavioural responses to an inflammatory challenge free from any individual variability explained by genetics. Our sample comprised animal with the same genetic background, at different ages. This allowed us to more easily highlight the physiological and behavioural effects of the experimental treatment on the cow’s age despite a small number of animals.

The LPS challenge triggered the expected inflammatory response. Pro-inflammatory cytokines (IL1-β, IL-6 and TNF-α) increased above basal levels from 3 to 12 hpi, and all cytokines except IL-6 fell back to basal level within 24 hpi. These results are in line with data already published on LPS challenge in cattle^19,24,26,27^. At the same time, the cows were in a febrile state characterized by less frequent RMR (< 1 cycle/2 min at 3 hpi), elevated RT (between 39.8 and 40.5 °C) and RR (between 26 and 43 breaths/min) at 3–6 hpi, and elevated HR (between 66 and 100 beats/min) for at least 24 hpi while RMR, RT and RR returned to baseline levels. Even though other LPS cows studies failed to show it^18,24^, the elevated HR was expected during the febrile state, as already reported after LPS injection in rats^52^. However, the fact that HR remained elevated for 24 h without returning to a basal level was more surprising. There may have been other mechanisms at play other than fever, such as constant nociceptive stimulation that could result in a marked and prolonged increase in HR rate. For example, dairy heifers subjected to oligofructose overload suffered subsequent acidosis then lameness and had elevated HR beyond 48 h^53^. This would suggest that in our study, the nociceptive component of the LPS injection has physiological consequences beyond the febrile phase, i.e. after 6 hpi. The release of cytokines and the febrile state confirmed LPS injection as a relevant frame for studying sickness-related behaviour in cattle. The resulting inflammatory response thus breaks down into two phases: the acute phase, in the first six hours, during which physiological, clinical and blood parameters reached a peak at 3 or 6 hpi; and a second phase lasting the following 18 h, during which these parameters decreased but without reaching the observed pre-challenge basal level for HR and IL-6.

The LPS injection likely caused inflammatory pain or at least severe discomfort for the cows that can be detected by sickness and pain-related behavioural changes during almost 24 hpi. Sickness behaviour is thought to reduce energy expenditure to sustain the metabolic cost of the fever response and thus facilitate recovery of homeostasis^54,55^. This interpretation is corroborated by the release of cortisol in blood from 3 to 12 hpi (+ 700% at 3 h compared to the pre-challenge level) and the increase in HR from 3 to 24 hpi, both of which reflect high metabolic activity. In our study, pain and discomfort were detected both by direct visual observations and by collar-attached accelerometers, but by different indicators at different times. Rapidly (from 3 to 6 hpi at least) and during the acute phase, the cows adopted specific postures (hunched back, lowered head, pressed tail) and facial expressions (strained nostrils, wide-open eyelids) that were detected by direct observations. The cows were also apathetic and less motivated to feed. Hunched back^35,56^, tail pressed against the udder^36^, and strained nostrils^37^ are clear postural modifications described in cattle suffering from pain. Simultaneously, the collar-attached accelerometers revealed that the cows modified their basic activities right after the LPS injection, as from the first hpi and for up to 10 hpi, the cows spent less time ingesting and more time without activity. To our knowledge, this study is the first to report early decrease in activity after an LPS injection in the jugular vein of cows to mimic systemic inflammation. Early decrease of activity has only been reported following LPS infusion but in the udder to mimic a local inflammation, where cows exposed to LPS spent significantly less cumulative amount of time eating from 3 h after the intramammary infusion^57^. After the acute phase (beyond 6 hpi), direct observations of the cows did not reveal changes in behaviour at 12 hpi. At 24 hpi, only two changes were identified (depressed feeding, and resting). Conversely, the accelerometers used here revealed that the decrease in activities in inflammation-challenged cows (more time without activity, more time standing, less time ruminating) continued beyond 6 hpi and lasted until at least 24 hpi. We expected to find changes in basic activities (e.g. feeding, ruminating, standing durations and motion) as such changes have been described in many cattle diseases (hypocalcaemia, ketosis, metritis, mastitis, lameness)^3^. Here, the cows no longer displayed postural and facial signs of pain at 12 and 24 hpi but beyond 6 hpi the basic activity changes detected by the accelerometers showed that they were still in discomfort. These behavioural changes were not identifiable by direct visual observations because they require prolonged monitoring. Here, these changes were associated with LPS injection, but using accelerometers to detect sickness may lack specificity because activity changes may result from a stressful event (e.g. changing pens or mixing batches)^58,59^. It would therefore be crucial to gather complementary information on the animal, chiefly through direct observation (e.g. facial or body expression, apathy) to confirm whether the animal is sick or in pain.

Due to the circadian rhythm of activity, the changes in cow activity detected by collar-attached accelerometers may differ depending on time of onset of inflammation (LPS injection). In more specific terms, the changes in activities captured by the accelerometers were visible either during the first 10 hpi or during the following 12 h period. These two periods of around 10–12 h not only differed in their timing after LPS injection but also in their circadian timing, i.e. daytime (from 9:30 AM to 9:30 PM) *vs*. night-time (from 9:30 PM to 9:30 AM). The spontaneous organization of behaviour during the day clearly interacts with the timing of the effects of LPS injection: for the first 12 h post-injection, the cows reduced their time spent feeding—at a time when they should be ingesting if not injected with LPS—and compensated by spending more time standing without activity. For the next 12 h, the cows spent less time ruminating—at a time they when should be ruminating if they had ingested sufficient feed just before—and more time standing up instead of lying down. The timing of these changes may have been different (not observed or delayed by 12 h) if the challenge had taken place at night instead of the morning: the decrease in feeding behaviour might have not been observed during the night or might have been observed on the following morning, and the decrease in ruminating might have been observed during the following night. The timing of the behavioural changes observed here therefore suggests that sensor-based systems would benefit from processing behavioural data per 12-h period rather than per 24-h period, preferably separating activities during day *vs.* night in order to avoid smoothing effects during a 24-h time-window and according to specific activities during these two different periods.

At 24 hpi, the basic activities of the cows—especially feeding and time ‘without activity’—had not completely returned to basal level, suggesting that sickness-related patterns of behaviour continued beyond that time. The persistence of elevated plasma IL-6, a cytokine clearly involved in the cerebral signalling of a depressive state^60^, the persistence of high HR (see above), and the presence of haptoglobin may all indicate that the inflammation had not resolved at 24 hpi. However, the long plasmatic half-life of haptoglobin makes it impossible to know whether the inflammation is still active. Moreover, administration of a LPS bolus is very different in terms of kinetics compared to a bacterial or viral infection where the microbes replicate, leading to the release of LPS and other inflammatory triggers, and does not resolve as rapidly as elimination of LPS by scavenger cells and detoxification mechanisms. It is therefore likely that the behavioural changes described here—including the changes detected during direct observations—would actually last much longer in the event of infectious diseases of various aetiologies.

Sickness and pain responses differed slightly between young and old cows. At the beginning of the inflammation, the direct visual observations identified specific signs related to discomfort and pain (apathy, changes in facial expression and body postures, reduction in feeding motivation-to-feed and ingestion) whatever the age of the cows. These signs of discomfort and pain then faded, and more quickly in young cows. However, the collar-attached accelerometers showed that the cows, and especially the older ones, were still inactive after the acute phase: they were standing up when they should have been lying down (during the night), and they displayed both prolonged inactivity during the night and day and prolonged lack of feeding leading to a prolonged lack of rumen motility. The old cows spent more time without activity and less time ruminating, especially at the end of the monitoring period. The greater amplitude of behavioural responses from old cows was further corroborated in the discriminant analysis that pooled all behavioural responses. This difference was similarly reported in mice after a viral challenge, where burrowing behaviour was more impaired in old than young mice^63^. Our results would suggest that older cows are slower to recover and that younger cows show more discreet patterns of sickness behaviour. These findings would further justify the need to combine detection strategies, i.e. a sensor-based system combined with clinical and behavioural examination to detect sick cows, even the youngest ones that have shown more discreet behavioural signs.

In conclusion, one can detect clinical signs of sickness and pain in cattle from direct observation rapidly after a controlled systemic challenge. Whatever the age of the cows, such signs develop quickly but may not last very long. A sensor-based system, such as the accelerometers used here, was able to detect changes in activities that occurred early during the inflammation process and for a prolonged period of time. These changes in activity were not specific to sickness and/or pain but were more marked in older cows than in younger cows. The combination of sensors and direct observations improved the detection of behavioural signs of sickness and pain earlier on and over the whole study period, even when direct signs were weak especially in young cows. These combinations could thus likely be crucial to detect sickness behaviour even the day after sickness has started. Because the short action of LPS injected intravenously is not totally mimicking a systemic disease, the benefit of the combination of sensors and direct observations to detect sickness and pain should be confirmed on natural cases of infection.

## Supplementary Information

Supplementary Information.

## Data Availability

The datasets used and/or analysed during the current study available from the corresponding author on reasonable request.
