# Supplementary material for: Combining accelerometers and direct visual observations to detect sickness and pain in cows of different ages submitted to systemic inflammation
Source: Sci Rep. 2023 Feb 3;13:1977. doi: 10.1038/s41598-023-27884-x (PMC9898231; doi:10.1038/s41598-023-27884-x)
Supplement: Supplementary file 1 — Supplementary Information. [file 41598_2023_27884_MOESM1_ESM.pdf]

SUPPLEMENTARY MATERIALS

Combining accelerometers and direct visual observations to detect sickness and pain in cows of different ages submitted to systemic inflammation

Dorothée Ledoux*[1], Isabelle Veissier[1], Bruno Meunier[1], Valérie Gelin[2,3], Christophe Richard[2], Hélène Kiefer[2,4], Hélène Jammes[2,4], Gilles Foucras[5], Alice de Boyer des Roches[1]

[1] Université Clermont Auvergne, INRAE, VetAgro Sup, UMR Herbivores, 63122 Saint-Genès-Champanelle, France

[2] Université Paris-Saclay, UVSQ, INRAE, BREED, 78350 Jouy-en-Josas, France

[3] UCEA, INRAE, Université Paris Saclay, 91630 Leudeville, France

[4] Ecole Nationale Vétérinaire d'Alfort, BREED, 94700 Maisons-Alfort, France

[5] IHAP, Université de Toulouse, ENVT, INRAE, 31076 Toulouse, France

Clinical parameters		Estimate	SE	t value
Rectal temperature (°C)	Fixed effects: coefficients of the model			
	(Intercept) T-1h, young	38.2	0.1	294.9
	T+3h	0.7	0.2	3.6
	T+6h	2.1	0.2	11.7
	T+12h	0.3	0.2	1.4
	T+24h	0.3	0.2	1.9
	Old (age)	0.2	0.2	1.1
	T+3h:Old	-0.2	0.3	-0.9
	T+6h:Old	-0.4	0.3	-1.5
	T+12h:Old	-0.1	0.3	-0.5
	T+24h:Old	0.1	0.3	0.5
	Random effects : standard deviations			
	Animal	0.0	0.1	-
	Residuals	0.1	0.3	-
Heart rate (beats/min)	Fixed effects: coefficients of the model			
	(Intercept) T-1h, young	57.0	4.2	13.4
	T+3h	18.0	6.0	3.0
	T+6h	12.0	6.0	2.0
	T+12h	29.5	6.0	4.9
	T+24h	30.5	6.0	5.1
	Old (age)	1.0	6.0	0.2
	T+3h:Old	6.5	8.5	0.8
	T+6h:Old	14.0	8.5	1.7
	T+12h:Old	-0.5	8.5	-0.1
	T+24h:Old	3.5	8.5	0.4
	Random effects : standard deviations			
	Animal	0.8	0.9	-
	Residuals	107.2	10.4	-
Respiratory rate (breaths/min)	Fixed effects: coefficients of the model			
	(Intercept) T-1h, young	10.3	1.8	5.7
	T+3h	29.0	2.4	12.2
	T+6h	11.0	2.4	4.6
	T+12h	3.0	2.4	1.3
	T+24h	4.0	2.4	1.7
	Old (age)	0.0	2.6	0.0
	T+3h:Old	-9.3	3.4	-2.8
	T+6h:Old	-3.3	3.4	-1.0
	T+12h:Old	-0.7	3.4	-0.2
	T+24h:Old	-0.2	3.5	-0.1
	Random effects : standard deviations			
	Animal	2.5	1.6	-
	Residuals	17.0	4.1	-
Rumen motility rate (cycles/2 min)	Fixed effects: coefficients of the model			
	(Intercept) T-1h, young	4.2	0.4	9.7
	T+3h	-2.3	0.5	-4.4
	T+6h	-1.2	0.5	-2.2
	T+12h	-0.3	0.5	-0.6
	T+24h	-1.2	0.5	-2.2
	Old (age)	-0.7	0.6	-1.1
	T+3h:Old	-0.2	0.8	-0.2
	T+6h:Old	0.7	0.8	0.9
	T+12h:Old	0.0	0.8	0.0
	T+24h:Old	-0.3	0.8	-0.4
	Random effects : standard deviations			
	Animal	0.3	0.5	-
	Residuals	0.9	0.9	-

Table S1 Coefficients of linear mixed effect models of clinical parameters in 6 old and 6 young Holstein cows one hour before (T-1 h) and at 3, 6, 12 and 24 h after injection of LPS intravenously (0.5 µg/kg of BW). Example of R formula rectal temperature lmer (RT) ~ Time + age + Time:age + (1 | animal).

Blood parameters		Estimate	SE	t value
LogCortisol (ng/mL)	Fixed effects: coefficients of the model			
	(Intercept) T-1h, young	1.6	0.1	12.9
	T+3h	0.6	0.1	5.1
	T+6h	0.4	0.1	3.6
	T+12h	0.1	0.1	1.0
	T+24h	0.0	0.1	-0.1
	Old (age)	-0.2	0.2	-1.1
	T+3h:Old	0.3	0.2	1.5
	T+6h:Old	0.1	0.2	0.6
	T+12h:Old	0.4	0.2	2.3
	T+24h:Old	0.4	0.2	2.2
	Random effects : standard deviations			
	Animal	0.3	0.5	-
	Residuals	0.9	0.9	-
Interleukin 1β (pg/mL)	Fixed effects: coefficients of the model			
	(Intercept) T-1h, young	3.5	2.8	1.2
	T+3h	17.3	3.1	5.6
	T+6h	13.6	3.1	4.4
	T+12h	9.8	3.1	3.2
	T+24h	3.1	3.1	1.0
	Old (age)	5.0	4.0	1.2
	T+3h:Old	-4.2	4.4	-1.0
	T+6h:Old	-1.8	4.4	-0.4
	T+12h:Old	-3.8	4.4	-0.9
	T+24h:Old	-3.9	4.4	-0.9
	Random effects : standard deviations			
	Animal	18.7	4.3	-
	Residuals	28.5	5.3	-

Table S2 Coefficients of linear mixed effect models of ~~clinica~~blood~~l~~ parameters in 6 old and 6 young Holstein cows one hour before (T-1 h) and at 3, 6, 12 and 24 h after injection of LPS intravenously (0.5 µg/kg of BW). Example of R formula for plasmatic cortisol: lmer (log10(plasmatic cortisol) ~ Time + age + Time:age + (1 | animal).

Blood parameters		Estimate	SE	t value
Log(Interleukin 6) (pg/mL)	Fixed effects: coefficients of the model			
	(Intercept) T-1h, young	0.5	0.2	2.6
	T+3h	2.0	0.2	9.5
	T+6h	2.0	0.2	9.5
	T+12h	1.4	0.2	6.7
	T+24h	0.5	0.2	2.4
	Old (Age)	0.3	0.3	1.0
	T+3h:Old	-0.1	0.3	-0.4
	T+6h:Old	-0.1	0.3	-0.2
	T+12h:Old	0.03	0.3	0.1
	T+24h:Old	0.3	0.3	0.9
	Random effects : standard deviations			
	Animal	0.1	0.4	-
	Residuals	0.1	0.4	-
Log(TNFα) (pg/mL)	Fixed effects: coefficients of the model			
	(Intercept) T-1h, young	1.9	0.2	10.2
	T+3h	2.6	0.2	11.4
	T+6h	1.6	0.2	7.2
	T+12h	0.9	0.2	3.9
	T+24h	0.5	0.2	2.0
	Old (age)	0.4	0.3	1.6
	T+3h:Old	-0.3	0.3	-0.9
	T+6h:Old	-0.1	0.3	-0.5
	T+12h:Old	-0.2	0.3	-0.7
	T+24h:Old	-0.04	0.3	-0.1
	Random effects : standard deviations			
	Animal	0.1	0.2	
	Residuals	0.2	0.4	
Haptoglobin (µg/mL)	Fixed effects: coefficients of the model			
	(Intercept) T-1h, young	0.00	14.9	0.00
	T+12h	1.0	20.9	0.05
	T+24h	237.9	20.9	11.4
	Old (age)	0.00	21.0	0.00
	T+12h:Old	-0.2	29.5	-0.01
	T+24h:Old	-65.8	29.5	-2.2
	Random effects : standard deviations			
	Animal	17.0	4.1	-
	Residuals	1307.2	36.2	-

Table S2 (continued) Coefficients of linear mixed effect models of ~~clinical~~ blood parameters in 6 old and 6 young Holstein cows one hour before (T-1 h) and at 3, 6, 12 and 24 h after injection of LPS intravenously (0.5 µg/kg of BW). Example of R formula for plasmatic haptoglobin: lmer (haptoglobin) ~ Time + age + Time:age + (1 | animal).

continuous monitoring of cow activities and position		Estimate	SE	t value
Time spent ingesting (min/h)	Fixed effects: coefficients of the model			
	(Intercept) T+1h, old, control	28.3	4.2	6.7
	Challenge (situation)	-18.3	6.0	-3.1
	Young (age)	-2.5	6.0	-0.4
	T+2h	15.0	6.0	2.5
	T+3h	-0.8	6.0	-0.1
	T+4h	-1.7	6.0	-0.3
	T+5h	2.5	6.0	0.4
	T+6h	-3.3	6.0	-0.6
	T+7h	-5.0	6.0	-0.8
	T+8h	2.5	6.0	0.4
	T+9h	9.2	6.0	1.5
	T+10h	15.0	6.0	2.5
	T+11h	-12.5	6.0	-2.1
	T+12h	-10.8	6.0	-1.8
	T+13h	-5.0	6.0	-0.8
	T+14h	-24.2	6.0	-4.0
	T+15h	-28.3	6.0	-4.7
	T+16h	-28.3	6.0	-4.7
	T+17h	-28.3	6.0	-4.7
	T+18h	-26.7	6.0	-4.5
	T+19h	-24.2	6.0	-4.0
	T+20h	-25.8	6.0	-4.3
	T+21h	-28.3	6.0	-4.7
	T+22h	-28.3	6.0	-4.7
	T+23h	-2.5	6.0	-0.4
	T+24h	-8.3	6.0	-1.4
	Challenge:Young:T+2h	0.0	12.0	0.0
	Challenge:Young:T+3h	-14.2	12.0	-1.2
	Challenge:Young:T+4h	0.8	12.0	0.1
	Challenge:Young:T+5h	18.3	12.0	1.5
	Challenge:Young:T+6h	-5.0	12.0	-0.4
	Challenge:Young:T+7h	-10.8	12.0	-0.9
	Challenge:Young:T+8h	8.3	12.0	0.7
	Challenge:Young:T+9h	35.8	12.0	3.0
	Challenge:Young:T+10h	35.0	12.0	2.9
	Challenge:Young:T+11h	6.7	12.0	0.6
	Challenge:Young:T+12h	-2.5	12.0	-0.2
	Challenge:Young:T+13h	-9.2	12.0	-0.8
	Challenge:Young:T+14h	-3.3	12.0	-0.3
	Challenge:Young:T+15h	0.0	12.0	0.0
	Challenge:Young:T+16h	0.0	12.0	0.0
	Challenge:Young:T+17h	0.0	12.0	0.0
	Challenge:Young:T+18h	1.7	12.0	0.1
	Challenge:Young:T+19h	4.2	12.0	0.3
	Challenge:Young:T+20h	2.5	12.0	0.2
	Challenge:Young:T+21h	0.0	12.0	0.0
	Challenge:Young:T+22h	0.0	12.0	0.0
	Challenge:Young:T+23h	11.7	12.0	1.0
	Challenge:Young:T+24h	-1.7	12.0	-0.1
	Random effects : standard deviations			
	Animal	0.0	0.0	-
	Residuals	107.0	10.3	-

Table S3 Coefficients of linear mixed effect models of continuous monitoring of cows activities and position in 6 old and 6 young Holstein cows at each time during 24h after injection of LPS intravenously (0.5 µg/kg of BW). Example of R formula for Time spent ingesting: lmer (time spent ingesting) ~ (Time + age + situation)^3 + (1 | animal).

continuous monitoring of cow activities and position		Estimate	SE	t value
	Fixed effects: coefficients of the model			
	(Intercept) T+1h, old, control	13.3	5.3	2.5
	Challenge (situation)	-0.8	7.4	-0.1
	Young (age)	-10.0	7.5	-1.3
	T+2h	-9.2	7.4	-1.2
	T+3h	-0.8	7.4	-0.1
	T+4h	-1.7	7.4	-0.2
	T+5h	-5.8	7.4	-0.8
	T+6h	-4.2	7.4	-0.6
	T+7h	7.5	7.4	1.0
	T+8h	-3.3	7.4	-0.5
	T+9h	-5.0	7.4	-0.7
	T+10h	-5.0	7.4	-0.7
	T+11h	18.3	7.4	2.5
	T+12h	0.8	7.4	0.1
	T+13h	-0.8	7.4	-0.1
	T+14h	25.8	7.4	3.5
	T+15h	21.7	7.4	2.9
	T+16h	18.3	7.4	2.5
	T+17h	23.3	7.4	3.2
	T+18h	16.7	7.4	2.3
	T+19h	15.8	7.4	2.1
	T+20h	27.5	7.4	3.7
	T+21h	18.3	7.4	2.5
	T+22h	31.7	7.4	4.3
	T+23h	1.7	7.4	0.2
Time spent ruminating (min/h)	T+24h	1.7	7.4	0.2
	Challenge:Young:T+2h	-5.0	14.8	-0.3
	Challenge:Young:T+3h	5.8	14.8	0.4
	Challenge:Young:T+4h	0.0	14.8	0.0
	Challenge:Young:T+5h	-7.5	14.8	-0.5
	Challenge:Young:T+6h	-4.2	14.8	-0.3
	Challenge:Young:T+7h	6.7	14.8	0.5
	Challenge:Young:T+8h	-13.3	14.8	-0.9
	Challenge:Young:T+9h	-25.8	14.8	-1.7
	Challenge:Young:T+10h	-34.2	14.8	-2.3
	Challenge:Young:T+11h	-10.0	14.8	-0.7
	Challenge:Young:T+12h	-16.7	14.8	-1.1
	Challenge:Young:T+13h	-16.7	14.8	-1.1
	Challenge:Young:T+14h	-10.8	14.8	-0.7
	Challenge:Young:T+15h	-19.2	14.8	-1.3
	Challenge:Young:T+16h	-5.8	14.8	-0.4
	Challenge:Young:T+17h	-16.7	14.8	-1.1
	Challenge:Young:T+18h	-12.5	14.8	-0.8
	Challenge:Young:T+19h	-18.3	14.8	-1.2
	Challenge:Young:T+20h	5.0	14.8	0.3
	Challenge:Young:T+21h	2.5	14.8	0.2
	Challenge:Young:T+22h	13.3	14.8	0.9
	Challenge:Young:T+23h	0.0	14.8	0.0
	Challenge:Young:T+24h	-3.3	14.8	-0.2
	Random effects : standard deviations			
	Animal	4.5	2.1	-
	Residuals	164.2	12.8	-

Table S3 (continued) Coefficients of linear mixed effect models of continuous monitoring of cows activities and position in 6 old and 6 young Holstein cows at each time during 24h after injection of LPS intravenously (0.5 µg/kg of BW).

continuous monitoring of cow activities and position		Estimate	SE	t value
Time without activity (min/h)	Fixed effects: coefficients of the model			
	(Intercept) T+1h, old, control	14.2	5.2	2.7
	Challenge (situation)	16.7	7.3	2.3
	Young (age)	5.8	7.4	0.8
	T+2h	-5.8	7.3	-0.8
	T+3h	-2.5	7.3	-0.3
	T+4h	-1.7	7.3	-0.2
	T+5h	-5.0	7.3	-0.7
	T+6h	-0.8	7.3	-0.1
	T+7h	-8.3	7.3	-1.1
	T+8h	-13.3	7.3	-1.8
	T+9h	-10.0	7.3	-1.4
	T+10h	-12.5	7.3	-1.7
	T+11h	-7.5	7.3	-1.0
	T+12h	5.0	7.3	0.7
	T+13h	-2.5	7.3	-0.3
	T+14h	-2.5	7.3	-0.3
	T+15h	10.8	7.3	1.5
	T+16h	14.2	7.3	1.9
	T+17h	9.2	7.3	1.3
	T+18h	5.8	7.3	0.8
	T+19h	10.0	7.3	1.4
	T+20h	-0.8	7.3	-0.1
	T+21h	14.2	7.3	1.9
	T+22h	-0.8	7.3	-0.1
	T+23h	-0.8	7.3	-0.1
	T+24h	3.3	7.3	0.5
	Challenge:Young:T+2h	25.0	14.6	1.7
	Challenge:Young:T+3h	12.5	14.6	0.9
	Challenge:Young:T+4h	5.8	14.6	0.4
	Challenge:Young:T+5h	6.7	14.6	0.5
	Challenge:Young:T+6h	11.7	14.6	0.8
	Challenge:Young:T+7h	15.0	14.6	1.0
	Challenge:Young:T+8h	10.0	14.6	0.7
	Challenge:Young:T+9h	-0.8	14.6	-0.1
	Challenge:Young:T+10h	14.2	14.6	1.0
	Challenge:Young:T+11h	9.2	14.6	0.6
	Challenge:Young:T+12h	26.7	14.6	1.8
	Challenge:Young:T+13h	34.2	14.6	2.3
	Challenge:Young:T+14h	16.7	14.6	1.1
	Challenge:Young:T+15h	29.2	14.6	2.0
	Challenge:Young:T+16h	12.5	14.6	0.9
	Challenge:Young:T+17h	24.2	14.6	1.7
	Challenge:Young:T+18h	5.0	14.6	0.3
	Challenge:Young:T+19h	18.3	14.6	1.3
	Challenge:Young:T+20h	-0.8	14.6	-0.1
	Challenge:Young:T+21h	5.0	14.6	0.3
	Challenge:Young:T+22h	-5.0	14.6	-0.3
	Challenge:Young:T+23h	1.7	14.6	0.1
	Challenge:Young:T+24h	7.5	14.6	0.5
	Random effects : standard deviations			
	Animal	3.1	1.8	-
	Residuals	160.8	12.7	-

Table S3 (continued) Coefficients of linear mixed effect models of continuous monitoring of cows activities and position in 6 old and 6 young Holstein cows at each time during 24h after injection of LPS intravenously (0.5 µg/kg of BW).

continuous monitoring of cow activities and position		Estimate	SE	t value
	Fixed effects: coefficients of the model			
	(Intercept) T+1h, old, control	58.3	6.5	9.0
	Challenge (situation)	-6.7	8.8	-0.8
	Young (age)	-3.3	9.2	-0.4
	T+2h	-6.7	8.8	-0.8
	T+3h	-5.0	8.8	-0.6
	T+4h	-3.3	8.8	-0.4
	T+5h	-9.2	8.8	-1.0
	T+6h	-11.7	8.8	-1.3
	T+7h	-11.7	8.8	-1.3
	T+8h	-5.0	8.8	-0.6
	T+9h	-1.7	8.8	-0.2
	T+10h	-5.0	8.8	-0.6
	T+11h	-25.0	8.8	-2.8
	T+12h	-19.2	8.8	-2.2
	T+13h	0.8	8.8	0.1
	T+14h	-46.7	8.8	-5.3
	T+15h	-51.7	8.8	-5.9
	T+16h	-40.0	8.8	-4.6
	T+17h	-50.8	8.8	-5.8
	T+18h	-41.7	8.8	-4.7
	T+19h	-37.5	8.8	-4.3
	T+20h	-49.2	8.8	-5.6
	T+210h	-49.2	8.8	-5.6
	T+22h	-40.8	8.8	-4.7
	T+23h	-15.0	8.8	-1.7
Time standing up (min/h)	T+24h	-5.0	8.8	-0.6
	Challenge:Young:T+2h	-16.7	17.6	-0.9
	Challenge:Young:T+3h	-21.7	17.6	-1.2
	Challenge:Young:T+4h	-18.3	17.6	-1.0
	Challenge:Young:T+5h	-6.7	17.6	-0.4
	Challenge:Young:T+6h	-15.0	17.6	-0.9
	Challenge:Young:T+7h	-30.8	17.6	-1.8
	Challenge:Young:T+8h	-22.5	17.6	-1.3
	Challenge:Young:T+9h	14.2	17.6	0.8
	Challenge:Young:T+10h	6.7	17.6	0.4
	Challenge:Young:T+11h	5.0	17.6	0.3
	Challenge:Young:T+12h	-5.8	17.6	-0.3
	Challenge:Young:T+13h	5.0	17.6	0.3
	Challenge:Young:T+14h	-21.7	17.6	-1.2
	Challenge:Young:T+15h	-29.2	17.6	-1.7
	Challenge:Young:T+16h	-7.5	17.6	-0.4
	Challenge:Young:T+17h	-30.0	17.6	-1.7
	Challenge:Young:T+18h	5.8	17.6	0.3
	Challenge:Young:T+19h	5.8	17.6	0.3
	Challenge:Young:T+20h	-14.2	17.6	-0.8
	Challenge:Young:T+21h	-13.3	17.6	-0.8
	Challenge:Young:T+22h	10.0	17.6	0.6
	Challenge:Young:T+23h	-3.3	17.6	-0.2
	Challenge:Young:T+24h	0.8	17.6	0.0
	Random effects : standard deviations			
	Animal	21.6	4.6	-
	Residuals	231.2	15.2	-

Table S3 (continued) Coefficients of linear mixed effect models of continuous monitoring of cows activities and position in 6 old and 6 young Holstein cows at each time during 24h after injection of LPS intravenously (0.5 µg/kg of BW).
